# Current Status and Challenges of US-Guided Radiofrequency Ablation of Thyroid Nodules in the Long Term: A Systematic Review

**DOI:** 10.3390/cancers13112746

**Published:** 2021-06-01

**Authors:** Stella Bernardi, Andrea Palermo, Rosario Francesco Grasso, Bruno Fabris, Fulvio Stacul, Roberto Cesareo

**Affiliations:** 1 Department of Medical Sciences, University of Trieste, 34149 Trieste, Italy; b.fabris@fmc.units.it; 2 U.C.O. Medicina Clinica, ASUGI (Azienda Sanitaria Universitaria Giuliano Isontina), Cattinara Hospital, 34149 Trieste, Italy; 3 Unità di Endocrinologia e Diabete, Policlinico Universitario Campus Bio-Medico, 00128 Roma, Italy; a.palermo@unicampus.it; 4 U.O.S. Radiologia Interventistica, Policlinico Universitario Campus Bio-Medico, 00128 Roma, Italy; r.grasso@unicampus.it; 5 S.C. Radiologia, ASUGI (Azienda Sanitaria Universitaria Giuliano Isontina), Maggiore Hospital, 34125 Trieste, Italy; stacul.fulvio@gmail.com; 6 U.O.S. Malattie Metaboliche, Ospedale Santa Maria Goretti, 04100 Latina, Italy; robertocesareo@libero.it

**Keywords:** US-guided minimally invasive techniques, radiofrequency ablation, RFA, benign thyroid nodules, thyroid cancer, DTC recurrences, PTMC, long term, follow-up, regrowth

## Abstract

**Simple Summary:**

Ultrasound (US)-guided minimally-invasive techniques, such as radiofrequency ablation (RFA) have emerged as an alternative treatment to surgery for benign and malignant thyroid nodules. Based on a systematic literature search, here we report the long-term outcomes of thyroid RFA. Available data show that US-guided RFA significantly reduced benign thyroid nodules and destroyed most PTMC, and this was generally maintained for at least 5 years after the initial treatment. Further studies addressing the risk of regrowth in patients with benign thyroid nodules, as well as the risk of recurrence in patients with PTMC are needed.

**Abstract:**

Background: US-guided minimally-invasive techniques, such as radiofrequency ablation (RFA) have emerged as an alternative treatment for benign and malignant thyroid nodules. This systematic review aims to provide an overview on the long-term outcomes of US-guided RFA in patients with benign and malignant thyroid nodules. Methods: We systematically searched PubMed/MEDLINE, EMBASE, and Scopus to identify articles reporting the outcomes of thyroid RFA after a follow-up of at least 3 years. Results: A total of 20 studies met the inclusion criteria and were included in the review. In patients with benign thyroid nodules, RFA significantly reduced nodule volume and this was generally maintained for the following 5 years. However, a small but not negligible proportion of nodules regrew and some of them required further treatments over time. In patients with malignant nodules, RFA has been used not only to treat differentiated thyroid cancer (DTC) neck recurrences, but also to treat papillary thyroid microcarcinoma (PTMC). In most patients with PTMC, RFA led to complete disappearance of the tumor. When it was compared to surgery, RFA was not inferior in terms of oncologic efficacy but it had a lower complication rate. However, RFA did not allow for final pathology, disease staging and accurate risk stratification. Conclusions: US-guided RFA significantly reduces benign thyroid nodules and destroys most PTMC, and this is generally maintained for at least 5 years after the initial treatment. Further studies addressing the risk of regrowths in patients with benign thyroid nodules, as well as the risk of recurrence in patients with PTMC are needed.

## 1. Introduction

Since the 80s, ultrasound (US) has played an increasingly important role in thyroid nodule assessment [1]. Beside thyroid nodule diagnosis, US has also been used for therapeutic purposes. In the last decade, US-guided minimally invasive techniques have been introduced into clinical practice as an alternative treatment for benign thyroid nodules as well as selected cases of differentiated thyroid cancer (DTC) [2,3,4,5,6]—such as neck recurrences or cases of low risk disease. These techniques go far beyond ethanol ablation, which has been the first technique to be introduced in the 90s [7], and they include laser, radiofrequency and microwave ablation, as well as HIFU [8]. A recent European survey on the use of US-guided minimally invasive techniques for thyroid nodules [9] showed that today RFA is the most frequently chosen, and so far it has been the most thoroughly assessed one.

US-guided RFA is an outpatient procedure, which is generally performed under local anesthesia [10,11]. It requires the US-guided insertion of a probe through the skin of the neck into the thyroid nodule. The probe tip generates heat which induces rapid heating and destruction of the target zone (i.e., one part of the nodule). Then, in order to treat an entire nodule, RFA is usually performed with the moving-shot technique, whereby the probe tip is sequentially moved from the medial to the lateral parts of the nodule, while it is slowly withdrawn towards the surface. Treatment is accompanied by the formation of coagulative necrosis, and over time, by fibrotic changes and progressive nodule shrinkage [5,11].

Several studies have demonstrated that this procedure is safe and effective, as it induces a significant reduction of benign thyroid nodule volume with improvement of local symptoms and cosmetic concerns [12,13], while in case of low-risk thyroid cancer, it is able to destroy the entire target zone [14,15,16,17]. In addition, in case of DTC neck recurrences or papillary thyroid microcarcinoma (PTMC), RFA has a similar efficacy and lower rate of complications than surgery [18,19,20]. Based on this background, accumulating evidence suggests that US-guided RFA could be used as a first line therapy not only for benign thyroid nodules but also for low-risk thyroid cancer.

Last year, one of the main focuses of the original articles that were published on thyroid RFA were its long-term outcomes [3,16,17,20,21,22,23], namely the volume reduction ratio and/or regrowths in patients with benign nodules and the recurrence rates in patients with malignant ones. Based on this, the aim of this systematic review was to provide an overview on these long-term outcomes of US-guided RFA in patients with either benign or malignant thyroid nodules, as well as to discuss its strengths and limitations.

## 2. Materials and Methods

The aim of this systematic review was to describe RFA long-term outcomes. Our primary outcome measure was volume reduction ratio. Our secondary outcome measures were regrowths in benign nodules and recurrence rates in malignant nodules. Based on the available literature on this topic, RFA long-term follow-up has been defined as a period of at least 3 years [24].

This systematic review was conducted following the PRISMA checklist. We conducted a systematic literature search on PubMed/Medline, EMBASE, Scopus to select all the studies reporting the follow-up of patients treated with RFA. The query included the terms “Radiofrequency”, “RFA”, “Thyroid” and “Follow-up”. To expand our search, references of the retrieved articles were also screened for other data. Further literature search was done based on these result and from the PubMed option “Related Articles”. The search was last updated on 28 February 2021.

**Figure 1:**
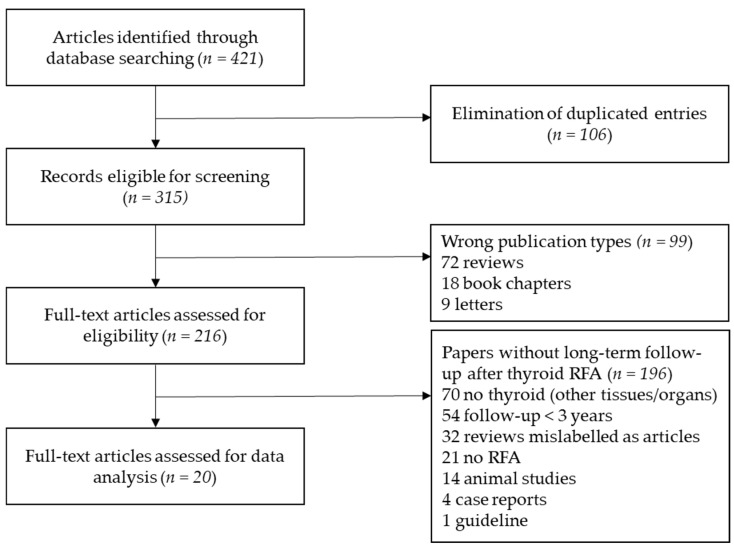
Stepwise procedure for study selection.

Figure 1 shows the stepwise procedure for study selection. We retrieved a total of 421 results. Studies were examined and selected for inclusion independently by two investigators (S.B. and A.P.) and a third one (R.C.) was consulted in case of controversy. Investigators were not blinded to authors, institutions, journals, or interventions while selecting studies. Inclusion criteria were as follows: (i) original studies; (ii) thyroid RFA; (iii) follow-up of at least three years. Exclusion criteria of studies were as follows: (i) studies not written in English; (ii) wrong publication types (reviews, meta-analysis, study protocols, case reports, letters, errata, conference proceedings, book chapters); (iii) wrong population (i.e., RFA performed on other tissues and organs); (iv) wrong outcome (i.e., follow-up shorter than three years). Studies were also excluded if relevant information regarding the study design or outcomes was unclear or if there was any doubt regarding duplicate publications. At the end of our qualitative analysis, we identified 20 studies. The paper by Kim et al. was included in our analysis because initial follow-up after RFA was 37.7 ± 10.8 months [18].

In order to assess the risk of bias of the included studies, we used the Cochrane Collaboration tool, namely the RoBANS [24]. Two authors (S.B. and B.F.) independently extracted data on study design, patient characteristics, RFA technique, volume reduction ratio, and follow-up and assessed risk of bias of included studies. Disagreements were resolved through discussion. The RoBANS assesses six domains of bias, specifically (D1) bias due to selection of participants, (D2) bias due to confounding variables, (D3) bias due to measurement of intervention, (D4) bias due to blinding of outcome assessment, (D5) incomplete outcome data follow-up, (D6) selective outcome reporting. In case of D1 we took into account if age, sex, nodule volume and cytology/pathology were specified. In case of D2 we took into account if RFA technique, number of RFA, and energy delivered were specified, in case of D3 we took into account if volume reduction ratio was specified, in case of D5 we took into account the number (proportion) of patient that were seen during follow-up. Traffic-light plots of risk of bias were designed using the robvis visualization tool [25].

## 3. Results

### 3.1. Long-Term Outcomes of RFA on Benign Thyroid Nodules

**Figure 2:**
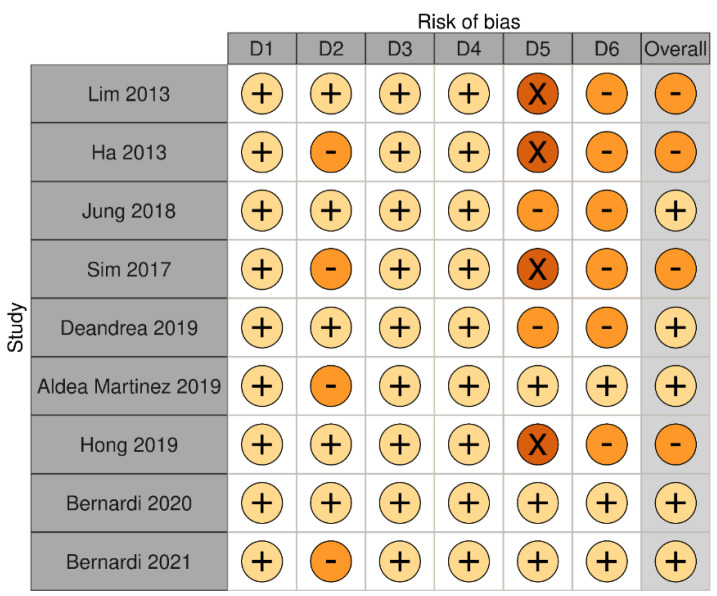
Bias assessment of studies on RFA long-term outcomes in patients with benign thyroid nodules. Risk of bias was classified as low (+) (pale yellow), unclear (-) (orange) or high (x) (brown). Risk of bias was based on the judgement of domains D1–D6. D1 is for selection of participants; D2 is for confounding variables; D3 is for measurement of intervention (VRR); D4 is for blinding of outcome assessment; D5 is for incomplete outcome data during follow-up; D6 is for selective outcome reporting. Risk of bias was judged unclear in D2 when number of RFA sessions or energy delivered were not specified. Risk of bias was judged high in D5 when the number of patients seen at specific follow-up time points was not specified. Risk of bias was judged unclear in D5 when the number of patients was lower than 80% of patients enrolled. Risk of bias in D6 was based on D5.

We identified a total of nine articles reporting the outcomes of RFA on benign thyroid nodules after a follow-up of at least three years (Table 1 and Table 2). Eight studies were retrospective and one study was prospective. Overall, these studies showed that RFA-induced volume reduction ratio ranged between 66.9% and 97.9% after three years from the procedure. Regrowth was observed in 4.1–24.1% of nodules. Bias assessment is reported in Figure 2.

The first retrospective study of this series was that of Lim et al. [26], who reported the outcomes of thyroid RFA on 111 patients (with 126 benign non-functioning nodules) after a follow-up duration of at least 3 years (mean follow-up length was 49.4 months, range 36–81 months). After a mean number of 2.2 ± 1.4 sessions, the Authors found that nodule volume was reduced by 70%, 90%, 90%, 90%, and 93.5% after 6 months, 1 year, 2 years, 3 years, and at last follow-up. This was associated with a significant amelioration of local symptoms and cosmetic concerns. Baseline nodule volume and solidity were independently associated with final volume reduction. Regrowth rate (i.e., an increase in nodule volume > 50% as compared with previous US examination) was 5.6%. Complication rate was 3.6% (4/111 patients), and they included voice change, brachial plexus injury, bruising and vomiting. The authors concluded that RFA is a safe and effective method and it can be used as a non-surgical treatment for patients with non-functioning benign thyroid nodules [26].

The subsequent studies confirmed most of these findings [26,28,30,31]. In particular, the efficacy and safety of RFA for the treatment of benign thyroid nodules have been confirmed by a prospective study [28] reporting RFA outcomes on 276 patients (with 276 nodules) treated on average with 1.3 sessions and followed for 46 months (range 15–79). In this study, nodule volume was reduced by 80%, 89%, 92%, and 95%, after 1, 2, 3, and 4 years from the procedure. Solidity and energy delivered were independent factors that predicted the final volume reduction. The overall complication rate was 5.1% (major complication rate was 1.1%), while side-effects occurred in 4.7% of the patients [28].

Contrary to the first studies evaluating the effects of multiple RFA sessions, Deandrea et al. reported the outcomes of one single session of RFA on 215 patients (with 215 nodules) followed for at least 3 years, showing that nodule volume was reduced by 67% at last follow-up. In this study, the nodules with a baseline volume < 10 mL showed the best response as their volume was reduced by 81% at last follow-up. This was associated with a significant amelioration of symptom and cosmetic scores. Regrowth, which was defined according to Lim et al. [26], occurred in 4.1% of nodules. There were no major complications, but only minor occurrences and side-effects, whose rates were 8.8% and 10%. Minor occurrences included hypotension, swelling, bruising, neck pain, fever and cough [30].

Other studies have extended these findings, showing that RFA does not modify thyroid function even in patients with previous lobectomy [34], such that the authors concluded that it can be considered as a first-line treatment for symptomatic benign thyroid nodules in order to preserve thyroid function. In addition, it has also been shown that RFA is effective and safe for non-functioning thyroid nodules in children and adolescents [32].

As opposed to the first long-term follow-up studies that focused primarily on the efficacy and safety of the technique, the most recent ones have brought up the issue of retreatment and regrowth [21,22,29], as defined by a recent proposal for standardization of terminology and reporting criteria [35]. Based on this proposal, nodule regrowth should be defined as a ≥50% increase compared to the minimum recorded volume measured at a given follow-up time point [35]. In the work by Sim et al., 52 patients (54 nodules) were followed for 39 months (range 13–87 months) after thyroid RFA. Mean volume reduction ratio (VRR) after the first procedure was 77% and 97.9% at last follow-up. Complication rate was 3.6% and side effect rate was 3.6%. In this study, 24.1% of the nodules regrew after a mean time of approximately 40 months, as assessed by the new definition [35]. The authors suggested that it is the residual vital volume increase what may cause/precede regrowth [29].

Consistent with these findings, we have recently evaluated the 5-year outcomes of one RFA and compared it to laser ablation (LA) in a cohort of 406 patients, 216 of whom were treated with RFA and the remaining 190 with LA [21]. It has to be noted that in this study, all patients were followed for at least five years after the procedure. Overall, RFA significantly reduced nodule volume, which decreased by 72%, 75%, 76%, 76% and 77% after 1, 2, 3, 4, and 5 years after the ablation. Regrowth was observed in 20% of patients treated with RFA (43/216), however only 12% of the patients were retreated, due to the fact that regrowth is not always associated with symptom relapse. After propensity score matching analysis, RFA was associated with greater volume reduction ratio (75% vs. 56%), technique efficacy (82% vs. 66%), as well as lower regrowth (17% vs. 34%) and retreatment rate (14% vs. 32%) as compared to LA. On logistic regression model analyses, energy delivered was the only parameter that was associated with technique inefficacy (cut-off was 1360 J/mL) and with regrowth (but the low AUC did not allow to elaborate any cut-off). Younger age, larger baseline volume, lower amount of energy (cut-off 918 J/mL) were associated with likelihood of retreatment [21].

Given that in the aforementioned work, we could not identify predictors of regrowth, we evaluated if the initial ablation ratio (IAR), which is a semiquantitative index that measures the amount of the ablated area, could predict regrowth [22]. For this reason, we analysed RFA outcomes on 78 patients (82 nodules) that were followed for 5 years after the procedure. Technique efficacy (i.e., volume reduction > 50% after 1 year) was achieved in 92% of patients, 23% of nodules regrew and 12% of nodules were retreated. Median IAR was 83%. IAR was significantly associated with technique efficacy, VRR, and with the likelihood of retreatment, but not with nodule regrowth [22]. In particular, an IAR > 49% was a good predictor of technique efficacy and an IAR > 73% was a good predictor of no retreatment in the five years following the procedure [22].

### 3.2. Long-Term Outcomes of RFA on Malignant Thyroid Nodules

**Figure 3:**
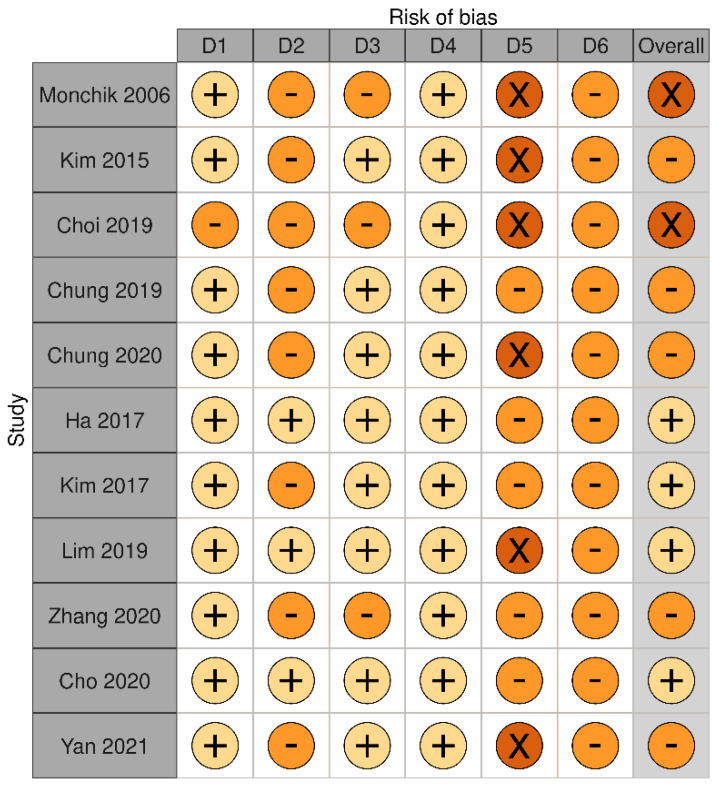
Bias assessment of studies on RFA long-term outcomes in patients with malignant thyroid nodule. Risk of bias was classified as low (+) (pale yellow), unclear (-) (orange) or high (x) (brown). Risk of bias was based on the judgement of domains D1-D6. D1 is for selection of participants; D2 is for confounding variables; D3 is for measurement of intervention (VRR); D4 is for blinding of outcome assessment; D5 is for incomplete outcome data during follow-up; D6 is for selective outcome reporting. Risk of bias was judged unclear in D1 when histology was not specified. Risk of bias was judged unclear in D2 when energy delivered was not specified. Risk of bias was judged unclear in D3 when VRR was not specified. Risk of bias was judged high in D5 when the number of patients seen at specific follow-up time points was not specified, but it was judged unclear if shorter follow-up time was >3 years. Risk of bias in D6 was based on D5.

We identified a total of 11 articles reporting the outcomes of RFA on malignant thyroid nodules after a follow-up of at least 3 years (Table 3, Table 4 and Table 5). All these studies were retrospective. Five studies evaluated RFA outcomes on differentiated thyroid cancer (DTC) neck recurrences, while the remaining six studies evaluated it on papillary thyroid microcarcinomas (PTMC), low risk papillary carcinoma, and small follicular neoplasms. Overall, these studies showed that RFA-induced volume reduction ratio ranged between 81.2% and 99.5% in DTC neck recurrences, between 98.5% and 100% in PTMC, and that it was 99.5% in follicular neoplasms. With respect to local recurrences, they ranged between 6.25% and 27% in DTC neck recurrences, and 0% and 4% in PTMC. Bias assessment is reported in Figure 3.

The use of RFA for the treatment of DTC neck recurrences dates back to the early 2000s [39], and it remains the only indication for thyroid RFA by some authoritative guidelines [40]. In 2006, Monchik et al. reported the outcomes of one or more RFA sessions on DTC neck recurrences in 16 patients that were followed for 41 months (range 10–68 months), showing that only one patient presented with a new recurrence in the neck. RFA caused laryngeal nerve injury in one patient and a 5-mm skin burn in another, as well as neck swelling and regional discomfort [36]. The authors concluded that RFA was a promising alternative to surgical treatment of DTC recurrence in patients with difficult reoperations [36]. Almost a decade after, Chung et al. confirmed that RFA is an effective and safe method for local control of DTC recurrences, as they reported that RFA reduced tumor volume by 99.5% and it induced its disappearance in 91.3% of patients after a mean follow-up of 80 months (range 60–114 months), with no delayed complications [37]. Local recurrences were seen in 27% of patients (8/29) and distant metastases in 6.9% of patients (2/29). Recently, the same authors have demonstrated that in DTC recurrences invading the airways [38], RFA reduced tumor volume by 81.2%, leading to its complete disappearance in 72% of the cases with an overall complication rate of 21.4%. The rate of volume reduction, tumor disappearance and complications were different as those reported previously, due to the inclusion of patients who were treated for curative and palliative purposes for recurrent tumors which were more likely to adhere or invade critical adjacent structures, increasing the complications rate during RFA [38].

A few papers have compared the efficacy and safety of RFA to surgery in the treatment of DTC neck recurrences. In particular, in the first study evaluating recurrences smaller than 2 cm, a total of 27 patients were treated with RFA, which induced a 98.4% volume reduction ratio and made 86% of lesions disappear. Overall, 12.5% of patients had a recurrence after 38 months. Recurrence free-survival rate was 93% and 88% at 1 and 3-year follow-up. After IPTW adjustement, 24 patients treated with RFA were compared to 40 patients treated with surgery and in the end the overall survival rate, as well as the 1- and 3-year recurrence-free survival rates, did not differ between groups, after a follow-up of almost 3 years (32 months) [18]. More recently, 96 patients with DTC neck recurrences were followed for 77 months after being treated with RFA. A total of 11.2% of them had a recurrence. The 3-year and 6-year recurrence-free survival rate was 91% and 89%. A subgroup of 70 patients was compared to 70 patients surgically treated. In this study, the recurrence-free survival rates were comparable between these groups, but the surgery group had a significantly higher rate of hypocalcemia [19].

Only very recently has RFA been used to treat low-risk PTMC. In the first study reporting RFA outcomes on low-risk small papillary thyroid carcinomas after a long-term follow-up, 66.7% lesions (4 out of 6) completely disappeared, while the remaining ones exhibited only small calcified residues [14]. No recurrences were reported after a mean follo-up of 4 years. More recently, in a cohort of 139 patients with 152 PTMC, RFA completely destroyed as many as 91.4% of ablated PTMC, while the remaining ones did not display any sign of regrowth after a mean follow-up duration of 39 months (6–104 months) [15]. When looking at the patients of this cohort with follow-up data of more than 5 years, 74 patients with 84 PTMC were selected for a subsequent study [17]. In these patients, RFA led to complete disappearance of all PTMC. There was no local tumor progression and no lymphnode or distant metastasis. There were four new PTMC in three patients in the remaining thyroid gland, which were ablated by RFA. The rate of minor complications was 4.1% (two hematomas and one first-degree skin burn) and that of major complications was 1.4% (one case of voice change) [17]. Likewise, in a cohort of 414 patients with unifocal PTMC, Yan et al. have recently demonstrated that RFA effectively reduced the target lesion by 98.8% and destroyed it in 88.4% of cases with no significant complications after a follow-up of 42 ± 12 months. In this study, the overall rate of local tumor progression was 3.62% (one persistent PTMC, four lymph node metastasis and 10 cases of new PTMC) [16]. It has to be noted that in a study retrospectively comparing RFA to surgery in case of unifocal PTMC, the Authors did not found any difference in terms of oncologic outcomes after 5 years of follow-up, but surgery took longer, had a longer hospitalization time, and was costlier than RFA [20].

## 4. Discussion

### 4.1. The Use of RFA to Treat Benign Thyroid Nodules

The advent of thyroid thermal ablations is likely to significantly change our conventional approach to thyroid nodules. In case of a benign thyroid nodule, patients are generally referred to surgery when they complain of symptoms or cosmetic concerns, while they are followed by US in case they do not. Thyroid thermal ablations, and RFA, represent a new therapeutic alternative in this conventional dichotomous scenario [3]. Randomized clinical trials have shown that RFA significantly reduces thyroid nodule volume and relieves from nodule-related clinical problems, such as local symptoms and cosmetic concerns [12,13]. The procedure does not affect thyroid function in euthyroid patients, and it is safe and extremely well tolerated. Our systematic review shows that RFA induces a volume reduction ratio ranging between 66.9% and 97.9% after 3 years from the procedure. Altogether, these data support the use of RFA to treat benign thyroid nodules, particularly when the target is a single, cold, benign, and symptomatic nodule [5,6,41]. Today, several scientific societies have included the use of minimally invasive techniques among the therapeutic options for symptomatic benign thyroid nodules [4,6,42]. In this new scenario, where observation, surgery and RFA are all viable options, we believe that RFA and surgery are not overlapping techniques, but they should be used to treat different types of nodules as well as different patients. Surgery for example is more effective than one RFA session for treating autonomously functioning nodules or very large nodules [10] (i.e., nodules with volumes > 20 mL). On the other hand, surgery may be unnecessary for treating small but symptomatic benign thyroid nodules that can be effectively managed with an outpatient procedure. This distinction applies also to the other minimally invasive technique, namely laser and microwave ablation or high-intensity focused ultrasound, whose choice depends also on local availability and physician expertise.

In case of benign thyroid nodules, one of the main differences between surgery and RFA is that surgery removes the entire nodule, while RFA reduces it (whereby it reduces patient symptoms). The follow-up studies that have been summarized in this review demonstrate that nodule volume reduction induced by RFA is generally maintained for (at least) 5 years. However, patients should be informed that in the vast majority of the cases a relatively small nodule persists. Some of the studies reviewed have tried to identify predictors of initial volume reduction and retreatment after RFA. These studies have demonstrated that baseline nodule volume, solidity, and applied energy predicted final volume reduction (and the likelihood of any retreatment). Deandrea et al. observed that the best response occurred in nodules with a volume < 10 mL, which were reduced by 82% and remained stable over time. By contrast, nodules with a volume between 10 and 20 mL and with a volume > 20 mL were reduced by 75% and 65% respectively [30]. This is consistent with the study by Lim et al., where patients with nodules < 10 mL were treated with 1.7 RFA sessions, while patients with nodules between 10 and 20 mL were treated with 2.8 RFA sessions and patients with nodules >20 mL were treated with 3.8 RFA sessions [26]. In our multicenter retrospective study, where RFA reduced nodule volume by 72% and 77% after 1 and 5 years from the procedure, and 12% of patients were retreated [21], large baseline volume was significantly associated with the likelihood of being retreated [21]. Of note, in this work, the baseline volume cut-off that predicted retreatment after RFA was 22.1 mL but it had an area under the curve indicating poor accuracy. By contrast, we found that a 1-year volume reduction <66% was a better predictor of nodule retreatment over time [21].

Energy delivered is another parameter that correlates with the volume reduction of benign thyroid nodules (as well as retreatments) [28,43,44,45]. In our multicenter study, an amount of energy delivered of 1360 J/mL was a moderately accurate predictor of technique efficacy (i.e., volume reduction >50% after 1 year) and an amount of energy < 918 J/mL was a good predictor of retreatment in the five years following the procedure [21]. Our data are consistent with the recent observation that delivering 756 J/mL, 1311 J/mL, 2109 J/mL was associated with a 50%, 75%, and 95% likelihood of technique efficacy [45].

Recent studies have shown that also the initial ablation ratio (IAR) [46] or the residual vital ratio (RVR) [47] might help predict volume reduction and/or retreatment after RFA [22,33,46,47]. The IAR is a semiquantitative index that measures the amount of ablation after 1–3 months from RFA and it is calculated as follows: IAR = (ablated volume/total volume) × 100 [46]. It has been shown that an IAR > 49% is a good predictor of technique efficacy and an IAR > 73% is a good predictor of no retreatment in the five years that follow the first procedure [22]. On the other hand, the RVR is a similar index that takes into account the viable volume instead of the ablated volume and it is calculated as follows: RVR = (viable volume/total volume) × 100 [33]. As opposed to the IAR, the greater the RVR the lower the likelihood of technique efficacy and the higher the likelihood of being retreated. We have recently found that a RVR > 73% was a moderately accurate predictor of technique inefficacy (i.e., volume reduction < 50% after 1 year) and a RVR > 60% was a good predictor of being retreated in the five years that follow the first procedure [33].

In contrast, the issue that remains to be fully understood is nodule regrowth [48,49]. There are several reasons that might explain such difficulty. First of all, nodule regrowth has been defined in different ways, which makes the existing follow-up studies difficult to compare. In addition, the majority of these studies have incomplete follow-ups, leading to an under or overestimate of regrowth. According to Mauri et al., regrowth is a ≥50% nodule volume increase compared to the minimum recorded volume measured at a given follow-up time point [35]. Taking into account this definition, regrowth seems to occur in 20% of patients treated with RFA after 5 years from initial treatment [21,29]. However, we do not know yet if this definition is too broad and might include regrowths that will never become clinically relevant problems. Second, most of the parameters related to volume reduction and retreatment (i.e., baseline volume, energy, IAR) have failed to predict nodule regrowth, apart from the RVR [47].

These data suggest that regrowth may be a distinct process from nodule shrinkage, and there might be other factors accounting for it, such as additional patient characteristics (ethnicity and iodine status for instance), as well as the nodule behaviour and/or technical issues. These technical issues include the operator experience, the lack of treatment of the nodule’s margins [29,48] or the lack of treatment of the feeding artery [50] and the draining vein, the latter of which is usually located at the nodule margins. Also the size and the position of the nodule influence the quality of an RFA treatment, given that the moving-shot technique (i.e., the probe repositioning) is tailored to the patient nodule and in large nodules or nodules whose location is close to critical structures it is difficult to treat the entire nodule [47]. In these cases, contrast-enhanced rather than non-enhanced ultrasound might provide useful information on the treatment outcome [51,52]. Additional classifiers based not only on clinical and US features, but also on molecular markers [53] and artificial intelligence might help predict treatment response as well as nodule behaviour over time [54].

### 4.2. The Use of RFA to Treat Differentiated Thyroid Cancer

The use of RFA is recommended by current guidelines in patients with suspected structural DTC neck recurrences [4,40]. In this circumstance, the use of RFA has been associated with volume reductions ranging from 55% to 95% and complete disappearance of metastatic foci in 40–90% of cases [36,55]. The few long-term follow-up studies that are available demonstrate that RFA is a promising alternative to surgical treatment in this setting, and this is confirmed by the fact that RFA and surgery have shown comparable recurrence-free survival rates [19].

By contrast, the use of RFA to treat PTMC or low-risk thyroid carcinoma has only recently been explored. The possibility to treat with RFA patients with PTMC or low-risk thyroid carcinoma unable or unwilling to undergo surgery has been introduced for the first time by the 2017 Korean Society of Thyroid Radiology guidelines [4]. Available studies show that when RFA is used to treat PTMC, it can effectively destroy the entire nodule, such that after 5 years from the procedure the ablation site reliably and consistently demonstrates no evidence of viable tumor [17]. In addition, Zhang et al. have recently shown that recurrence rates did not differ between RFA and surgery and that RFA had a lower complication rate and a higher quality of life than surgery [20]. Based on this ground, RFA could become a third therapeutic option in a scenario where the choices are either active surveillance or surgery.

The basic goals of initial therapy for patients with differentiated thyroid cancer are not only to improve overall and disease-specific survival and reduce the risk of persistent/recurrent disease, but also to permit accurate disease staging and risk stratification [40]. Surgery is the only approach that can remove the primary tumor and permit accurate staging and risk stratification of the disease [40].

Nevertheless, the ATA guidelines have recently introduced the possibility of chosing an active surveillance approach in case of patients with very low risk tumors, such as PTMC without clinically evident metastases or local invasion and no cytologic/genetic evidence of aggressive disease [40]. This is based on the observation that loco-regional recurrence rates, distant recurrence rates, and mortality rates do not differ between patients with PTMC who underwent surgery and those who were only followed up [40]. Ito et al. followed 1235 patients with PTMC for an average 5–6 years (up to 15 years), and 1.5% of them showed lymph node metastases, 3.5% showed progression to clinical disease, 4.6% showed tumor size enlargement [56], indicating that PTMC have an indolent nature.

Having said that, if RFA became a third option for PTMC, it would have some limitations/drawbacks. As compared to surgery, for example, RFA does not allow for identification of additional foci of PTMC as well as micrometastases in the central neck compartment [3]. As compared to active surveillance, on the other hand, if patients with PTMC were treated with RFA, it is not entirely clear how to monitor them, given that during active surveillance, the primary biomarker to signal that surgical intervention is warranted is the change in size of the primary tumor [23]. On this ground, we believe that studies on larger cohorts of patients followed for a longer period of time are needed to understand if the risk of disease recurrence and metastatic spread after RFA will not differ from surgery or the active surveillance approach [23].

### 4.3. What Do the Guidelines State about the Use of RFA

Based on some of these and other pioneering studies, several authoritative international societies have included RFA among the treatment modalities for thyroid nodule management and/or have established specific recommendations for the use of RFA.

In 2016, the American Association of Clinical Endocrinologists (AACE) introduced the possibility to use laser or radiofrequency ablation for the treatment of solid or complex thyroid nodules that progressively enlarge or are symptomatic or cause cosmetic concerns, after repeating fine-needle aspiration for cytology confirmation [42]. On the other hand, in the same years, the American Thyroid Associations (ATA) suggested the use of RFA only in case of structural neck recurrences of DTC (particularly in case of lesions measuring > 10 mm), in high-risk surgical patients or patients refusing additional surgery [40]. Here RFA was also taken into account in case of liver, lung, and bone metastases in patients iodine-refractory metastatic DTC [40].

A few scientific societies and national working groups have developed also specific practice guidelines on the use of thermal ablations for the treatment of thyroid nodules. The principal and most recent statements have been issued by the following groups: the Korean Society of Thyroid Radiology (KSThR) [4], the Italian working group on minimally invasive treatments of the thyroid (MITT) [5], interdisciplinary working groups of German and Austrian professional associations [57,58], and the European Thyroid Association (ETA) [6,59]. The principal indication is the treatment of non-functioning benign nodules that are symptomatic, although the use of RFA can be taken into account in case of autonomously functioning thyroid nodules when radioiodine or surgery are contraindicated or unwanted. The Korean and Austrian statements include also the treatment of local recurrences of iodine-refractory thyroid carcinoma, while the use of RFA in “low-risk” papillary PTMC is taken into account by the KSThR as well as by the ETA [59], while it remains an area “under discussion” [58] for the Austrian working group.

As far as benign thyroid nodules are concerned, this systematic review shows that the best RFA results have been generally obtained in nodules with baseline volume <10– 20 mL [21,26,30]. Therefore, we believe that RFA should be used to treat preferentially symptomatic benign lesions if their volume does not exceed 20–30 mL (unless they are cystic or predominantly cystic). Our results highlight also that the best RFA results have been generally obtained when energy delivered was >1300 J/mL. Therefore, guidelines should emphasize the importance of energy delivered as a key to successful treatment.

As far as malignant thyroid nodules are concerned, our systematic review show that recurrence-free survival rates do not differ between RFA and surgery in patients with DTC recurrences, which is in line with the concept that RFA can be used to treat DTC recurrences in patients in high-risk surgical patients or patients refusing additional surgery. On the other hand, we believe that further (and longer) studies are needed before extending RFA to “low-risk” papillary microcarcinomas. In particular, the lack of definite histopathological information in the absence of diagnostic surgery will represent a significant impediment to its use in this setting.

### 4.4. Strenghts and Limitations

This systematic review addresses what is currently seen as an open question [58], namely the regrowth/recurrence rates in the long-term follow-up after RFA. The limitations include the fact that regrowth has been defined in many ways, studies are heterogeneous in terms of number of treatments and energy delivered, and -most importantly- the majority of them has incomplete follow-ups and in many cases the number of patients seen over time is not specified.

## 5. Conclusions

Current scientific literature indicates that RFA is an effective treatment of benign thyroid nodules. The ideal target appears to be a single, cold, benign, and symptomatic thyroid nodule, with a baseline volume below 20 mL. In this nodule, RFA should deliver more than 1300 J/mL, in order to achieve a satisfactory volume reduction and avoid retreatments in the following 5 years. Nodule regrowth remains poorly understood, and patients should be followed after initial procedure. RFA represents a therapeutic option in patients with DTC neck recurrences that have a high surgical risk, or refuse additional surgery. On the other hand, it might become a treatment for PTMC in the future, particularly once the issue of accurate staging and risk stratification of the disease as well as removal of micrometastases have been resolved.

## Figures and Tables

**Table 1:** Main findings of studies on RFA long-term outcomes in patients with benign thyroid nodules.

Study	Main Findings
Lim 2013 [26]	RFA was effective in reducing nodule volume and nodule-related problems such as symptoms and cosmetic concerns (mean VRR was 93.4% at last follow-up). Regrowth rate was 5.6%.
Ha 2013 [27]	RFA reduced nodule volume by 87.2% at last follow-up and it did not affect thyroid function in patients with previous lobectomy.
Jung 2018 [28]	Nodule volume was reduced by 80.3% after 1 year (*n* = 276) and by 95.3% after 5 years (*n* = 6). Solidity and applied energy predicted final volume reduction.
Sim 2017 [29]	RFA reduced nodule volume by 97.9% at last follow-up. Regrowth was observed in 24.1% of the nodules.
Deandrea 2019 [30]	The VRR that was found at 1 year (63% in 197/215 patients) was maintained at 5 years (67% in 71/215 patients). The best results were obtained in nodules with baseline volume < 10 mL. A total of 4.1% of nodules regrew.
Aldea Martinez 2019 [31]	RFA reduced nodule volume by 76.8% after 3 years (in 24/24 patients).
Hong 2019 [32]	RFA reduced nodule volume by 92.1% at last follow-up in children and adolescents with no complications.
Bernardi 2020 [21]	After propensity score matching, RFA was associated with greater 5-year VRR (75% vs. 56%) and technique efficacy (82% vs. 66%), as well as lower regrowth (17% vs. 34%) and retreatment rate (14% vs. 32%) as compared to LA. Young age, large volume, low 1-year VRR, and low energy delivered were associated with retreatments.
Bernardi 2021 [22]	RFA reduced nodule volume by 79% after 5 years (in 78/78 patients). IAR was significantly associated with technique efficacy, VRR, and the likelihood of retreatment but not with regrowth. IAR cut-off were >49% for technique efficacy and >73% for no retreatment.

IAR initial ablation ratio, LA is for laser ablation, RFA is for radiofrequency ablation, VRR is for volume reduction ratio.

**Table 2:** Main characteristics of studies on RFA long-term outcomes in patients with benign thyroid nodules.

Study	Design	Patients/Nodules *	Age (yrs)	Sex (F%)	Volume (mL)	Diameter (mm)	RFA (n)	Energy (J/mL)	VRR (%)	Follow-Up (Months)
Lim 2013 [26]	Retrospective	111/126	37.9 ± 10.6 (9–69)	91	9.8 ± 8.5 (2–43)	33 ± 10 (20–60)	1–6	2936 ± 1995 (271–9943)	93.5 ± 11.7 (17–100)	49.4 ± 13.6 (36–81)
Ha 2013 [27]	Retrospective	11/14	44.2 (30–64)	100	9.7 ± 36.3 (0.9–57.6)	29 ± 24 (15–60)	n.s	n.s.	87.2	43.7 ± 30.7 (7–92)
Jung 2018 [28]	Prospective	276/276	46.3 ± 12.8 (15–79)	88	14.2 ± 13.2 (1.1–80.8)	38 ± 11 (19–80)	1–2	4161 ± 2993 (656–22,031)	95.3 ± 4.3 (88.5–100)	60
Sim 2017 [29]	Retrospective	52/54	44.1 ± 13.2 (20–78)	91	14 ± 12.7 (3.1–56.6)	38 ± 11 (19–77)	1–?	n.s.	97.9	39.4 ± 21.7 (13–87)
Deandrea 2019 [30]	Retrospective	215/215	66# (60–88)	85	20.9# (15–33)	n.s.	1	2210# (1400–3080)	66.9#	60
Aldea Martinez 2019 [31]	Prospective	24/24	50.2 ± 13.6	83	36.3 ± 59.8 (0.7–231.6)	n.s.	1–?	1180 ± 716	76.8 ± 15.9	36
Hong 2019 [32]	Retrospective	15/15	15.7 ± 2.3 (12–19)	71	14.6 ± 13.3 (1.6–49.8)	37 ± 11 (20–56)	1–5	3153 ± 2065 (782–7504)	92.1 ± 11.4	36.9 ± 21.7 (6–69)
Bernardi 2020 [21]	Retrospective	216/216	57# (17–87)	75	17.2# (0.4–179)	n.s.	1	1398# (176–2410)	77.1# (−34.5–100)	60
Bernardi 2021 [33]	Retrospective	78/82	59.5# (18–86)	76	11.3# (0.4–54.6)	23.5# (17.3–30.1)	1	n.s.	79	60

Continuous variables are reported as mean ± SD [min—max] or median# [min—max]. “n” is for number, “n.s.” is for not specified, VRR is for volume reduction ratio (at last follow-up). # Median values, * The number of patients/nodules refers to the groups treated with RFA (before matching).

**Table 3:** Main findings of studies on RFA long-term outcomes in patients with malignant thyroid nodules.

Study	Main Findings
**DTC Neck Tecurrences**	
Monchik 2006 [36]	No recurrent disease was detected at the treatment site in 14/16 patients.
Kim 2015 [18]	After IPTW adjustement, the 3-year recurrence-free survival rate after RFA was comparable to surgery (92.6% vs. 92.2%).
Choi 2019 [19]	After PSM, the recurrence-free survival rate after RFA was comparable to surgery (98% vs. 95%).
Chung 2019 [37]	RFA reduced DTC recurrences by 99.5% at 5 years and 91.3% of them disappeared. Local recurrences were seen in 27% of patients.
Chung 2021 [38]	RFA reduced nodule volume by 81.2% and made disappear 124/172 recurrences (72.1%) after 48 months.
**Small Follicular Neoplasm**	
Ha 2017 [34]	RFA reduced the volume of follicular neoplasms by 99.5% after 5 years. 8 out of 10 lesions (80%) disappeared.
**Low-Risk Papillary Carcinomas/PTMC**	
Kim 2017 [14]	RFA reduced the volume of papillary carcinoma by 98.5%. 4 out of 6 lesions (66.7%) disappeared. There were no recurrences.
Lim 2019 [15]	RFA led to complete disappearance of 91.4% of PTMC, and the remaining PTMC did not regrow. There were no recurrences.
Zhang 2020 [20]	RFA was not inferior to surgery in terms of recurrences (1.1% vs. 1.3%). The surgery group had a higher complication rate and a lower quality of life than the RFA group.
Cho 2020 [17]	RFA resulted in complete disappearance of all ablated tumors, with no local tumor progression, no lymph-node or distant metastases. 3 patients developed 4 new cancers (4%).
Yan 2021 [16]	VRR was 98%. A total of 88.4% of tumors disappeared. Local tumor progression rate was 3.62%. Recurrence rate was 3.4%.

DTC is for differentiated thyroid cancer, IPTW is for inverse probability of treatment weights, PSM is for propensity score matching, PTMC is for papillary thyroid microcarcinoma, RFA is for radiofrequency ablation, VRR is for volume reduction ratio.

**Table 4:** Studies on RFA long-term outcomes in patients with DTC neck recurrences.

Study	Design	Patients/Nodules *	Age (yrs)	Sex (F%)	Volume (mL)	Diameter (mm)	RFA (n)	E (J/mL)	VRR (%)	Recur-rence	Follow-Up (Months)
**DTC Neck Recurrences**											
Monchik 2006 [36]	Retrospective	16/16	53 (28–84)	75	n.s.	17 (8–40)	1–6	n.s.	n.s.	1/16 (6.25%)	40.7 (10–68)
Kim 2015 [18]	Retrospective (vs. surgery)	27/36	42.4 ± 10.3	74	n.s.	21.1 ± 1.01	1–2	n.s.	98.4 ± 6.2 (77–100)	3/26 (11.5%)	37.7 ± 10.2
Choi 2019 [19]	Retrospective (vs. surgery)	96/115	47.4 ± 14.1	72	n.s	10 ± 8	1–3	n.s.	n.s.	12/96 (12.5%)	76.9 ± 23
Chung 2019 [37]	Retrospective	29/46	51.8 ± 15 (21–84)	59	0.25 ± 0.4 (0.001–2.3)	8.4 ± 4.7 (3.1–21)	1–3	n.s.	99.5 ± 2.9 (81–100)	8/29 (27%)	80 ± 17.3 (60–114)
Chung 2021 [38]	Retrospective	119/172	50.7 ± 16 (14–83)	72	0.4 ± 1.4 (0.001–12.6)	9 ± 6 (3–41)	1–5	n.s.	81.2 ± 55.7	n.s.	47.9 ± 35.4 (6–128)

Continuous variables are reported as mean ± SD [min–max]. DTC is for differentiated thyroid cancer, “n” is for number, “n.s.” is for not specified, RFA is for radiofrequency ablation, VRR is for volume reduction ratio (at last follow-up). * The number of patients/nodules refers to the groups treated with RFA (before matching).

**Table 5:** Studies on RFA long-term outcomes in patients with low-risk thyroid cancers.

Study	Design	Patients/Nodules *	Age (yrs)	Sex (F%)	Volume (mL)	Diameter (mm)	RFA (n)	E (J/mL)	VRR (%)	Recur-rence	Follow-Up (Months)
**Small Follicular Neoplasm**											
Ha 2017 [34]	Retrospective	10/10	45 ± 10.5 (27–74)	100	0.6 ± 0.4 (0.2–1.6)	14 ± 3 (10–19)	1–2	9245 ± 5409 (3976–19,332)	99.5 ± 1 (97–100)	0/10 (0%)	66.4 ± 5.1 (60–76)
**Low-Risk Papillary Carcinomas/PTMC**											
Kim 2017 [14]	Retrospective	6/6	72 (64–79)	66	0.3 ± 0.2 (0.05–0.4)	9.2 (6–13)	1–2	n.s.	98.5 ± 3.3 (92–100)	0/6 (0%)	48.5 ± 12.3 (36–65)
Lim 2019 [15]	Retrospective	133/152	46 ± 12 (19–79)	85.7	0.03 ± 0.04 (0.001–0.3)	4.3 ± 1.4 (3–10)	1–2	3169 ± 1423 (600–11,550)	100	0/133 (0%)	39 ± 25 (6–104)
Zhang 2020 [20]	Retrospective (vs surgery)	94/94	45 ± 10.8	74.5	0.17 ± 0.23	6.14 ± 2.54	1	n.s.	n.s.	1/94 (1.1%)	64.2 ± 2.8
Cho 2020 [17]	Retrospective	74/84	46 ± 12	89	0.02 (0.001–0.23)	4 (3–9.9)	1–2	185,237 (13,088–4,716,379)	100	3/74 (4%)	72 ± 18 (60–124)
Yan 2021 [16]	Retrospective	414/414	43.6 ± 9.8 (18–73)	78	0.09 ± 0.08 (0.001–0.5)	5.22 ± 1.59 (2–10)	1–?	n.s.	98.8 ± 6.4 (50–100)	15/414 (3.62%)	42.1 ± 11.9 (24–69)

Continuous variables are reported as mean ± SD [min–max]. “n” is for number, “n.s.” is for not specified, PTMC is for papillary thyroid microcarcinoma, RFA is for radiofrequency ablation, VRR is for volume reduction ratio (at last follow-up). * The number of patients/nodules refers to the groups treated with RFA (before matching).
